# Impact of Environmental Modifications on the Ecology, Epidemiology, and Pathogenesis of *Plasmodium falciparum* and *Plasmodium vivax* Malaria in East Africa

**DOI:** 10.4269/ajtmh.21-1254

**Published:** 2022-10-13

**Authors:** Guiyun Yan, Ming-Chieh Lee, Guofa Zhou, Ai-Ling Jiang, Teshome Degefa, Daibin Zhong, Xiaoming Wang, Elizabeth Hemming-Schroeder, Wolfgang R. Mukabana, Arlene E. Dent, Christopher L. King, Kuolin Hsu, James Beeson, John I. Githure, Harrysone Atieli, Andrew K. Githeko, Delenasaw Yewhalaw, James W. Kazura

**Affiliations:** ^1^ Program in Public Health, University of California at Irvine, Irvine, California;; ^2^ Center for Hydrometeorology and Remote Sensing, Department of Civil and Environmental Engineering, University of California at Irvine, Irvine, California;; ^3^ Department of Medical Laboratory Sciences, Institute of Health, Jimma University, Jimma, Ethiopia;; ^4^ Center for Global Health & Diseases, Case Western Reserve University, Cleveland, Ohio;; ^5^ Department of Biology, University of Nairobi, Nairobi, Kenya;; ^6^ Burnet Institute for Medical Research and Public Health, Melbourne, Victoria, Australia;; ^7^ Tom Mboya University College, Homa Bay, Kenya;; ^8^ School of Public Health and Community Development, Maseno University, Kisumu, Kenya;; ^9^ Centre for Global Health Research, Kenya Medical Research Institute, Kisumu, Kenya;; ^10^ Tropical and Infectious Diseases Research Center, Jimma University, Jimma, Ethiopia

## Abstract

Food insecurity, recurrent famine, and poverty threaten the health of millions of African residents. Construction of dams and rural irrigation schemes is key to solving these problems. The sub-Saharan Africa International Center of Excellence for Malaria Research addresses major knowledge gaps and challenges in *Plasmodium falciparum* and *Plasmodium vivax* malaria control and elimination in malaria-endemic areas of Kenya and Ethiopia where major investments in water resource development are taking place. This article highlights progress of the International Center of Excellence for Malaria Research in malaria vector ecology and behavior, epidemiology, and pathogenesis since its inception in 2017. Studies conducted in four field sites in Kenya and Ethiopia show that dams and irrigation increased the abundance, stability, and productivity of larval habitats, resulting in increased malaria transmission and a greater disease burden. These field studies, together with hydrological and malaria transmission modeling, enhance the ability to predict the impact of water resource development projects on vector larval ecology and malaria risks, thereby facilitating the development of optimal water and environmental management practices in the context of malaria control efforts. Intersectoral collaborations and community engagement are crucial to develop and implement cost-effective malaria control strategies that meet food security needs while controlling malaria burden in local communities.

## INTRODUCTION

African governments have embarked on major programs during the past two decades to develop and strengthen the continent’s water resource development to ensure food security and alleviate poverty. These efforts have required large financial investments in land-use modifications, such as the construction of dams to generate hydroelectric power^1^^–^^3^ and agricultural irrigation canals to enhance subsistence and cash crop productivity.^4^^–^^7^ Malaria control and elimination are challenged by these environmental modifications and ongoing demographic trends, such as increasing urbanization, deforestation, and human migration, which can affect the transmission of malaria and other vector-borne infectious diseases.^8^

Although many dam and irrigation projects appear to increase malaria transmission, there are also examples where similar projects have had no impact on transmission or even resulted in reduced malaria prevalence.^9^^,^^10^ Thus, the impact of dams and irrigation on malaria transmission is variable and likely depends on the ecology of local mosquito vectors, the epidemiological setting, and preexisting control measures. Predicting the impact of dams and irrigation requires a deeper understanding of the ecological and hydrological consequences of agricultural development, sedimentation, and siltation on vector larval ecology and malaria transmission dynamics in parallel with the changing risk of malaria in local communities. Behavioral changes, species shifts, and increased insecticide resistance in malaria vectors, as observed widely across Africa, pose additional challenges. The overarching goal of our International Center of Excellence for Malaria Research (ICEMR) is to assess the impact of human-induced environmental modifications such as dam construction and irrigation on malaria epidemiology, transmission, pathogenesis, and immunology. More broadly, our intent is to generate scientifically sound evidence from field studies and develop analytic tools that inform local and national efforts to control and eliminate malaria in Kenya and Ethiopia. This article highlights the progress of our ICEMR efforts toward these goals since its inception in 2017.

## STUDY COUNTRIES AND SITES

We selected densely populated regions of Kenya and Ethiopia as the sites for our ICEMR research. Mountainous western Kenya and southwestern Ethiopia have high rainfall, and have experienced major investments in dam construction and irrigation of agricultural farms for food production in the recent past.^11^^–^^13^ Several features of malaria epidemiology are similar in both countries and include 1) increasing insecticide resistance and outdoor biting behavior of malaria vectors;^14^^–^^16^ 2) a high incidence of uncomplicated and severe malaria;^17^ 3) a high prevalence of blood-stage infection in asymptomatic children and adults (i.e., “subclinical malaria”);^18^^–^^20^ 4) spatial heterogeneity of transmission intensity and vectorial systems;^21^^–^^24^ 5) decreasing but not eliminated malaria transmission resulting from widespread implementation of artemisinin-based combination therapy, long-lasting insecticidal nets (LLINs), and indoor residual spraying of insecticides (IRS); and 6) a changing age profile of symptomatic malaria such that not just children younger than 5 years, but also older children and adults, experience uncomplicated and severe malaria.^25^^,^^26^

The sub-Saharan ICEMR also addresses gaps in knowledge with respect to *Plasmodium vivax* infection in Africa. Ethiopia ranks third worldwide with respect to the annual number of *P. vivax* malaria cases.^27^ Yet, little is known about its relapse pattern, the effectiveness of low-dose primaquine treatment recommended to eliminate liver-stage hypnozoites in an outpatient field setting, or the impact of erythrocyte Duffy blood group negativity on symptomatic *P. vivax* infection and naturally acquired immunity.

**Figure 1.**
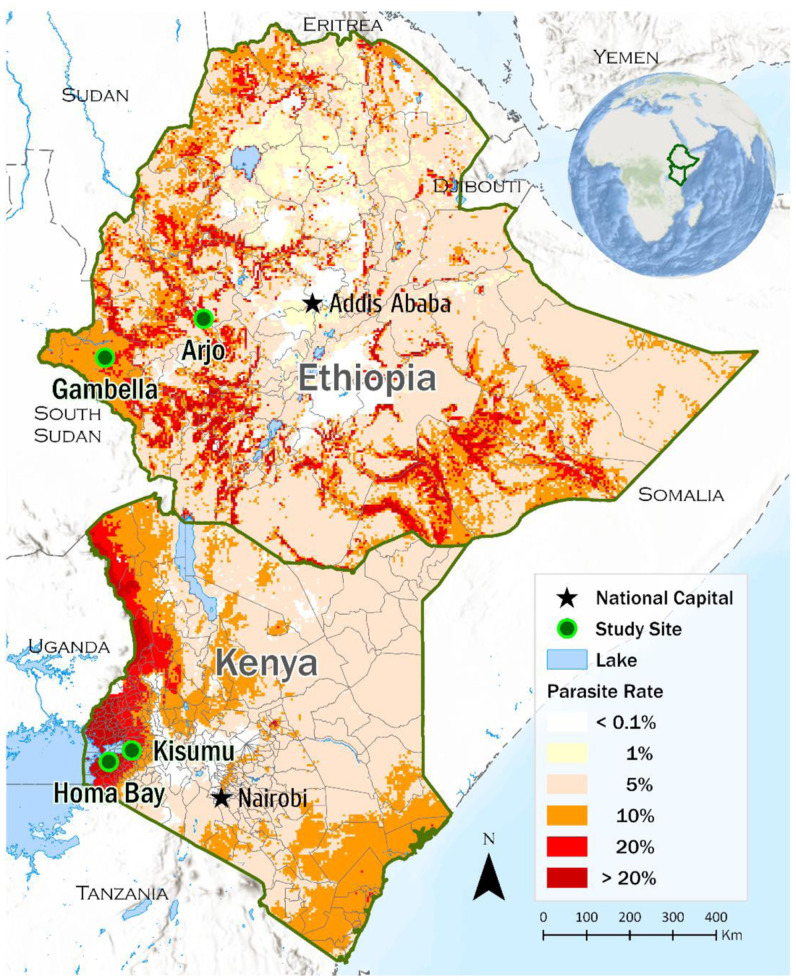
International Center of Excellence for Malaria Research study sites in relation to malaria endemicity in Ethiopia and Kenya. Malaria endemicity is indicated by the population-adjusted estimate of *Plasmodium falciparum* infection rate in 2- to 10-year-old children (PfPR_2–10_) for Kenya and the sum of *P. falciparum* plus *Plasmodium vivax* infections for Ethiopia. The map was adapted from the 2019 Malaria Atlas Project database.^28^

The ICEMR has two study sites in Ethiopia (Arjo and Gambella), and two sites in western Kenya (Homa Bay County and Kisumu County) (Figure 1^28^). Arjo is an irrigated site for a sugarcane plantation owned by the Ethiopian government. Gambella is a large-scale irrigation site for rice farming owned by international investors. The Kimira Oluch irrigation scheme in Homa Bay, Kenya, is a mixed-crop irrigation site constructed with the support of the Kenyan government, with funding from the African National Development Bank. Maize, vegetables, fruit, and rice are cultivated. All the irrigation schemes are gravity fed by dams located 10 to 20 km upstream. The Kisumu County ICEMR site in Kenya has no irrigation and practices traditional rain-fed agriculture. The four study areas differ considerably with respect elevation, the variety of crops that are cultivated, and the predominant mosquito vector and malaria parasite species (Table 1).

**Table 1:** Ecological and epidemiological characterization of the International Center of Excellence for Malaria Research study sites

Country	Site name	Elevation, m	Ecological setting	Predominant crops	Malaria endemicity	Predominant vectors	Predominant malaria species
Ethiopia	Gambella	480–550	Dam and irrigation	Rice	Stable	*Anopheles arabiensis*, *Anopheles pharoensis*	*Plasmodium falciparum*
	Arjo	1,250–1,500	River and irrigation	Sugarcane	Unstable	*An. arabiensis*, *Anopheles coustani*	*Plasmodium vivax*
Kenya	Homa Bay	1,150–1,500	River and irrigation	Mixed crops	Stable	*An. arabiensis,* *An. coustani*	*P. falciparum*
	Kisumu	1,150–1,650	Rainfed agriculture	Maize	Stable	*Anopheles gambiae, Anopheles funestus*	*P. falciparum*

## IMPACT OF DAMS AND IRRIGATION ON MALARIA VECTOR ECOLOGY AND TRANSMISSION

At the Arjo irrigation site in Ethiopia, we observed more diverse anopheline larval habitats, 2-fold greater anopheline-positive habitats, and 17% greater anopheline larval abundance compared with nearby nonirrigated areas.^29^ Consistent with larval ecology findings, adult mosquito surveillance found that the abundance of *Anopheles gambiae *s.l. collected indoors and outdoors was 10.8- and 14.5-fold greater in irrigated areas than nonirrigated areas, respectively.^30^ Life table studies showed that the mean survival time of female *An. gambiae* adults in the irrigated and nonirrigated areas was 38 days and 31 days, respectively.^31^ Longer survival of adult mosquitoes in irrigated areas enhances vectorial capacity. Similar trends in *Anopheles* species diversity and density were observed in Gambella, Ethiopia.^32^ The overall transmission intensity at the Homa Bay irrigation site measured by the entomological inoculation rate was about 3-fold greater in the irrigated area than in surrounding nonirrigated areas. Interestingly, molecular barcoding analysis revealed extensive new *Anopheles* cryptic species and genotypes that harbor unusually high *Plasmodium* sporozoite rates with a preference to feed on humans.^33^ The presence of such diversified malaria-transmitting *Anopheles* species could contribute to increased risk of year-round malaria transmission, and thus complicate disease prevention and control.

Intensive agriculture activities in irrigated areas are often associated with greater pesticide use. As such, pesticide use for agricultural pest control poses additional selection pressure for insecticide resistance in malaria vectors and may contribute to the emergence of insecticide resistance in *An. gambiae* in irrigated areas of Homa Bay, Kenya,^34^ and Arjo and Gambella, Ethiopia.^35^ Thus, vector surveillance and control in areas with anticipated or ongoing environmental modifications should examine both emerging cryptic species and rapidly increasing insecticide resistance.

## INTEGRATION OF HYDROLOGICAL MODELING WITH MALARIA TRANSMISSION MODELING

**Figure 2.**
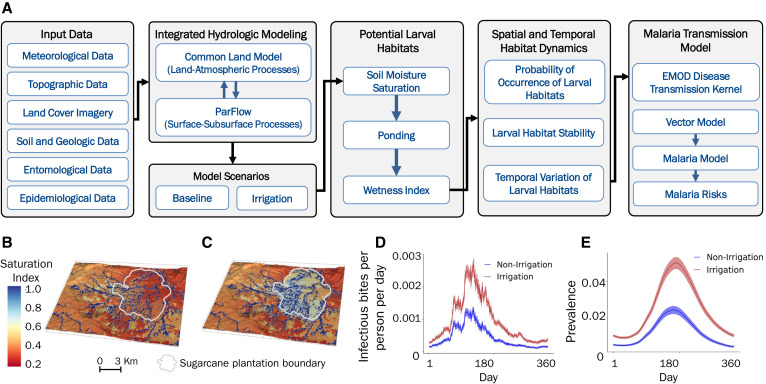
Integration of hydrological model with remote sensing and malaria transmission model to predict the impact of irrigation and water management on malaria vectors and disease risks within a sugarcane plantation in Arjo, Ethiopia. (**A**) Flow chart of modeling processes. (**B**) Predicted dry-season soil moisture saturation distribution in the absence of irrigation. (**C**) Dry-season soil moisture saturation distribution when the sugarcane plantation was irrigated. (**D**) Predicted impact of irrigation on daily entomological inoculation rate. (**E**) Impact of irrigation on malaria parasite prevalence. EMOD = Epidemiological MODeling software. Figure was adapted from Jiang et al.^36^

To simulate the effect of irrigation on water ponding and larval habitat formation, we used the ParFlow hydrological model to estimate surface water distribution and derive malaria vector aquatic habitats. ParFlow is an integrated watershed model that simulates complex topography and coupled land-surface processes under various climate and environmental factors such as meteorology, soil properties, land use and land cover, and topography (Figure 2A^36^). Model simulation results showed that irrigation increased significantly the probability of occurrence of larval habitats during both the dry and rainy seasons (Figure 2B and C). Larval habitat stability was prolonged, with a significant shift from semipermanent to permanent habitats. The high-risk period for larval breeding (June–September) was also extended by 2 months as a result of irrigation. Spatial distribution and duration of larval habitats predicted by the model a priori helps in identifying larval habitats and high-risk areas that are critical to vector larval source management.^37^ By integrating the hydrological model with a malaria transmission model, the impact of irrigation on malaria transmission (Figure 2D) and disease risk (Figure 2E) can be modeled. This integrated approach can be used to develop water management strategies that meet the water need of the crops and reduce malaria transmission.

## MALARIA SEROLOGY AND FUNCTIONAL ANTIBODY STUDIES

**Figure 3.**
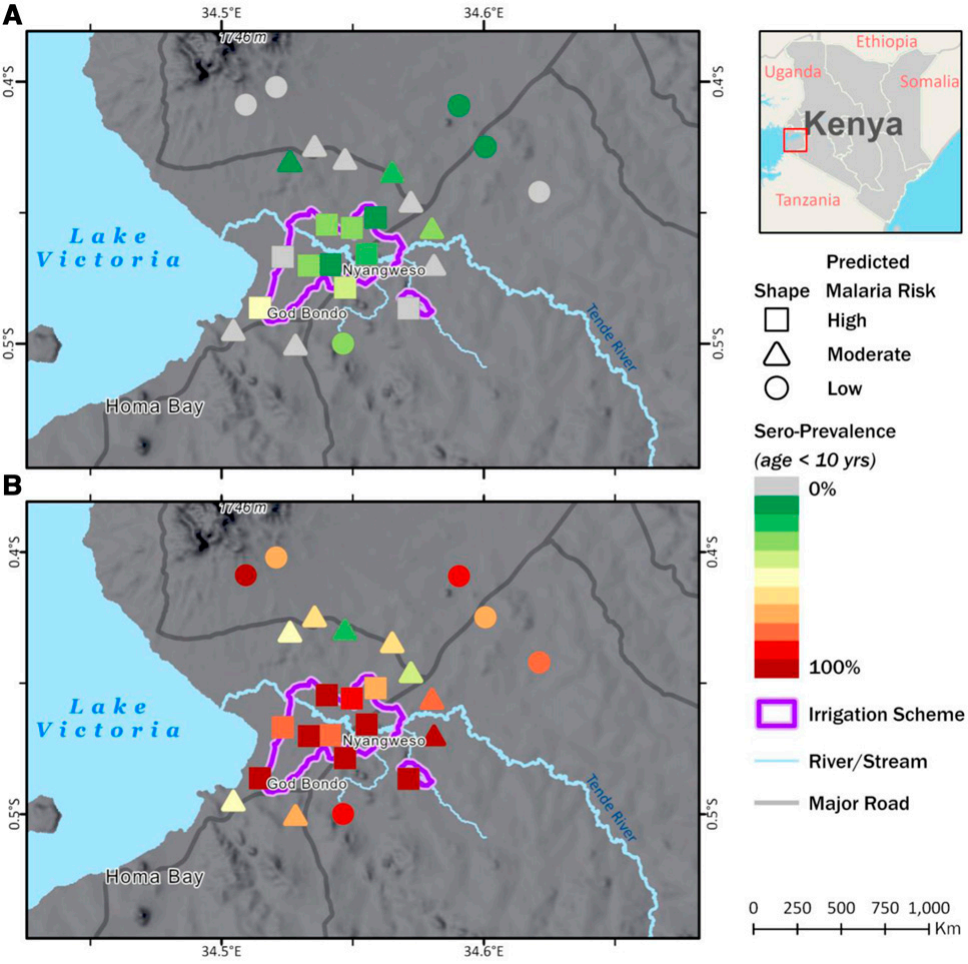
Spatial distribution of seropositivity for *Plasmodium falciparum* circumsporozoite protein and apical membrane antigen 1 among 1- to 10-year-old children in Homa Bay, Kenya, according to predicted malaria risk based on distance to irrigation area and elevation. (**A**) Circumsporozoite protein IgG antibody prevalence. (**B**) apical membrane antigen 1 IgG antibody prevalence.

We have used blood-stage and pre-erythrocytic *Plasmodium falciparum* antigens to form the “backbone” for our serology assays using multiplexed Magpix technology (Luminex Corp, Austin, TX). As shown in Figure 3A, we observed a significantly greater prevalence of seropositivity for the pre-erythrocytic protein circumsporozoite protein among 1- to 10-year-old children residing in the irrigation area than those residing outside the irrigation area. Notably, circumsporozoite protein antibodies have a relatively short half-life and are indicative of infection within the past year.^38^ In contrast, antibodies to blood-stage antigen apical membrane protein 1 (Figure 3B), which is highly immunogenic with a slow antibody decay rate, were less heterogeneous with respect to child residence in irrigation or nonirrigation sites. Ongoing studies are using this approach to identify geospatial hotspots of malaria exposure and to track the temporal dynamics of malaria risk in areas where IRS with a new neonicotinoid insecticide is anticipated to be implemented in 2023.

Another area of focus of our ICEMR immunity studies is in defining the functional mechanisms by which antibodies to sporozoites and gametocytes mediate protection, and understanding how varying levels of *P. falciparum* exposure affect the acquisition and maintenance of functional antibody activity. Adults residing in high-transmission areas with life-long malaria exposure in western Kenya have significant humoral immunity to sporozoites,^39^ but the role of these antibody functions in protective immunity is currently unclear. Antibodies to gametocytes may act in two main ways: 1) by targeting gametocyte-infected red blood cells to promote clearance and thereby reduce transmission, and 2) by targeting antigens expressed on the gametocyte plasma membrane. When a mosquito takes a blood meal containing gametocytes, these antibodies have the potential to neutralize or promote killing of gametocytes and gametes in the mosquito midgut. At the high-transmission Kisumu County site, we found that antibodies to gametocyte-infected red blood cells were rare and occurred at very low levels. In contrast, antibodies to gametocytes were prominent among adults and some children.^40^

## IMPACT OF IRRIGATION AND DAMS ON MALARIA BURDEN

We have conducted longitudinal monitoring of malaria burden in both Kenya and Ethiopia. Preliminary analysis of data collected at Homa Bay from 2020 to 2021 found a 2.5-fold increase in clinical malaria incidence in the irrigated villages compared with the surrounding nonirrigated villages. To determine the impact of environmental changes from irrigation on the molecular force of infection, defined as the number of new parasite clones acquired over time, we enrolled a prospective cohort of residents from irrigated and surrounding nonirrigated villages. We found that the molecular force of infection detected by *P. falciparum cpmp* amplicon deep sequencing was approximately 7.5-fold greater in the irrigated area than in the nonirrigated area. Furthermore, in a cross-sectional study in Homa Bay, we used sequencing data and population genetic analysis to reveal that the irrigated area, with the highest transmission, provided a source of parasite infections for the surrounding nonirrigated area.^41^

**Figure 4.**
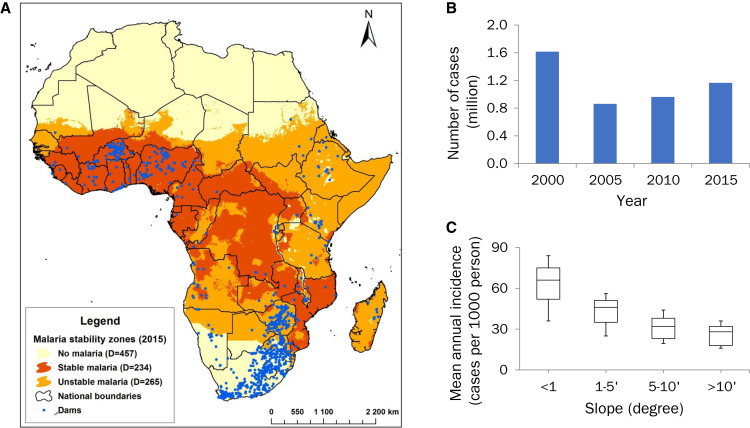
Impact of large dams in Africa on malaria burden. (**A**) Distribution of large dams in relation to malaria stability in 2015 in sub-Saharan Africa. (**B**) Number of annual malaria cases attributable to large dams in sub-Saharan Africa. (**C**) Relationship between malaria incidence and reservoir slope, showing a lower slope at the shoreline having greater impacts on malaria. Figure was adapted from Kibret et al.^42^

African countries have been building large dams for the past two decades to generate a reliable source of electricity and to improve water security and flood control. In a recent meta-analysis based on the information in the World Register of Dams database,^42^ we found that the number of large dams increased from 884 in 2000 to 919 in 2015 (Figure 4A^42^). During the same period of time, the number of people living within approximately 5 km of large dam reservoirs, where malaria risk is the greatest, increased from approximately 14.4 million to 18.7 million. A total of 700,000 to 1.6 million annual malaria cases were attributed to large dams in sub-Saharan Africa (Figure 4B). The slope of the dam reservoir shoreline is the most important predictor of a dam’s impact on malaria transmission (Figure 4C). Reservoirs with a gentle slope at the shoreline tend to enhance transmission relative to those with a steep slope. A gentle slope results in poor drainage, promoting the persistence of surface water bodies and formation of stable pools that are favorable habitats for mosquito breeding. In contrast, a steeper slope facilitates drainage and reduces the likelihood that pools will form for periods long enough for mosquitoes to complete their aquatic development.

## VECTOR SURVEILLANCE TOOL DEVELOPMENT AND EVALUATION

**Figure 5.**
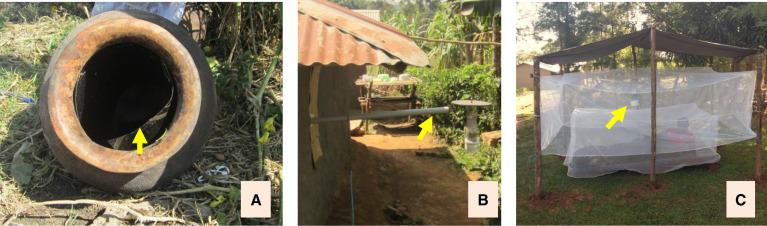
New outdoor resting and host-seeking malaria vector surveillance tools developed and evaluated at International Center of Excellence for Malaria Research study sites. (**A**) Sticky clay pot, with arrow showing the waterproof paper coated with Tangle-Trap sticky substance. (**B**) Human-odor baited CDC light trap, with arrow showing the polyvinyl chloride pipe that connects to a human sleeping room to move human odors from indoors to an outdoor mosquito-catching station via a fan. (**C**) Human-baited double net/CDC light trap combination, with arrow showing the CDC light trap. Figure was adapted from Degefa et al.^43^^,^^44^

Surveillance of malaria vectors is critical to measure transmission intensity and evaluate the impact of control interventions. In most African countries, vector surveillance activities have mainly relied on sampling host-seeking and indoor resting mosquitoes. The most commonly used methods for sampling host-seeking vectors are human-landing catches and CDC light traps, whereas indoor resting vectors are sampled by pyrethrum spray catches and use of Prokopack or backpack aspirators. During the past decade of widespread use of LLINs and IRS, there has been an increasing shift in vector species composition from anthropophagic, endophilic to zoophagic, exophilic sibling species. Yet, outdoor resting and biting vector sampling is seldom included in routine surveillance because of the lack of standardized and efficient traps. We developed and evaluated several alternative vector surveillance tools suitable for outdoor collection of mosquitos at our ICEMR study sites.^43^^,^^44^ These include sticky pots, human odor–baited CDC light traps, and the human-baited double-net/CDC light traps combined with sticky pots (Figure 5A^43^^,^^44^). The sticky variant of a clay pot (the pot is covered with waterproof black paper coated with Tangle-Trap sticky substance) has been used previously to collect outdoor resting *Anopheles* mosquitoes.^45^ Addition of the sticky substance allows for continuous collection of all mosquitoes that come to rest in the pot, rather than only those that are resting at the time a standard clay pot is retrieved. Unlike some alternative trapping methods, sticky pots are made using locally available clay pots and do not require a battery. In outdoor mosquito collections in western Kenya, sticky pots collected 52.5% more female *An. gambiae* mosquitoes than clay pots (6.1 versus 4.0 mosquitoes per trap per night, *P* < 0.01) and 38.3% fewer mosquitoes than CDC light traps (6.1 versus 9.9 mosquitoes per trap per night, *P* < 0.01).^43^ Human odor–baited CDC light traps (Figure 5B) consist of a CDC light trap baited with human odor pumped from an individual sleeping indoors. It captured 2.2 times as many malaria vectors as the regular CDC light trap (1.1 versus 0.5 females per trap per night, *P* < 0.05) at our ICEMR study site in Arjo, Ethiopia. Human-baited double-net/CDC light trap combinations (Figure 5C) are an improved modification of the previously designed double-net trap integrated with a CDC light trap. Mosquitoes attracted to the human bait are collected by setting a CDC light trap between the two nets. Field testing in Ethiopia showed that these traps caught six times as many malaria vectors as the CDC light trap (3.3. versus 0.5 females per trap per night, *P* < 0.001), similar to the number of mosquitoes collected by the traditional human-landing catch method.^44^ These new tools are potentially cost-effective and user friendly for outdoor resting and host-seeking malaria vector surveillance. Compared with the commonly used CDC light trap, sticky pots can be scaled up at a fraction of the cost. Because mosquito collection using the human odor–baited double-net/CDC light trap combination or human odor–baited CDC light trap uses human odor as an attractant, they do not directly expose human collectors to potentially infective mosquitoes.

## EVALUATION OF LONG-LASTING LARVICIDE FORMULATION

Larval control is a promising intervention because it targets both indoor and outdoor biting mosquitoes. It complements first-line indoor-based vector control tools such as LLINs and IRS. However, currently available biolarvicide formulations have short residual efficacy and, consequently, larval control incurs a high operational cost as a result of the requirement for frequent re-treatment of larval habitats. Formulations of biolarvicides with long-lasting effects are highly desirable, but their effectiveness is unknown. We evaluated the effectiveness of a slow-release microbial larvicide based on *Bacillus thuringiensis israelensis* and *Bacillus sphaericus* in reducing habitat productivity, adult malaria vector abundance, and clinical malaria incidence in western Kenya.^46^ No pupae emerged from treated habitats during the first 2 months of microbial larvicide application, and *Anopheles* pupal density decreased by more than 85% in the next 3 to 5 months. The mean densities of indoor and outdoor biting mosquitoes decreased by 76% to 82% and 67% to 75%, respectively. Slow-release microbial larvicide application in larval habitats did not alter the abundance or diversity of aquatic nontarget organisms cohabiting sites with mosquito larvae.^47^

## EVALUATION OF MALARIA DIAGNOSIS AND SURVEILLANCE METHODS

Data collected from health facilities and community-based malaria infection surveys are used to stratify malaria risk. Different vector control strategies are deployed according to risk stratification. Low-risk malaria zones in the central highlands of Kenya and several regions of Ethiopia are targeted for elimination.^48^^,^^49^ Consequently, sensitive diagnostic methods to enhance surveillance system detection of clinical malaria cases and sub-microscopic asymptomatic infections are needed. We evaluated whether there is added value in using a recently developed ultra-sensitive rapid diagnostic test (RDT) that has a lower limit of detection of *P. falciparum* parasitemia than the conventional RDT in western Kenya. Two malaria RDTs (Alere™ ultra-sensitive RDT and CareStart™ PfHRP2-RDT [conventional RDT]) were examined using samples from febrile patients seeking health services. We found that ultra-sensitive RDT detected a 35.8% positivity rate, which was significantly greater than the 30.4% positivity rate detected by conventional RDT and 20.9% by blood smear microscopy. These results suggest that ultra-sensitive RDTs can improve malaria diagnosis in these health-care settings. Furthermore, the high prevalence of histidine-rich protein 2 and 3 gene (*pfhrp2/3*) gene deletions in the Horn of Africa, including Ethiopia, warrants careful monitoring of *pfhrp2/3* gene deletions and selection of quality-ensured RDTs that are not based on detection of the PfHRP2 protein.^50^

Kenya was the first country in sub-Saharan Africa to deploy an online health information system powered by District Health Information Software 2 for malaria data management.^51^ All Kenyan districts and selected health facilities are connected to the District Health Information Software 2 national server to manage and analyze aggregated malaria data. In contrast, Ethiopia has adopted a one-plan–one-budget–one-report policy, and implemented an electronic health management information system for malaria data management as an integral part of health system monitoring.^52^ Although these information systems provide data for rapid assessment of malaria risk and burden, the quality of malaria case data collected at local health facilities needs to be validated in terms of its diagnostic accuracy. Clinical malaria incidence detected by passive case detection at community health facilities at the ICEMR study site in Kisumu County, Kenya, was 77% less than the incidence detected by active case detection in an ICEMR cohort of residents from the same area. This discrepancy resulted in part from a proportion of febrile residents who did not attend local health facilities, but who purchased antimalarial drugs from community drug shops (31.2%) or sought herbal medicines from local providers (3.5%).^53^ These findings suggest that data from online health information systems should be used with caution when interpreted to reflect population-level malaria risk and burden. Increased community awareness of malaria symptoms, and health-seeking behavior education are clearly important areas for improvement at our ICEMR study sites.

## MONITORING UNCOMPLICATED AND SEVERE MALARIA

ICEMR study sites in western Kenya historically and currently have the greatest *P. falciparum* transmission intensities in the country. We evaluated the impact of various vector interventions on community clinical malaria burden using passive case detection for uncomplicated malaria in selected public and private hospitals. Data collected to date indicate that clinical malaria continues to be an important cause of febrile illness in children and adults. For example, we observed a total of 2,282 uncomplicated cases of malaria (children and adults presenting with febrile illness, *P. falciparum*—positive blood smear, and no signs of severe malaria) in a sub-district hospital in Kisumu County in a 12-month period spanning 2018 to 2019, including 1,130 cases in children younger than 5 years. During the same time period, there were 32 cases of cerebral malaria diagnosed based on criteria set forth by the Kenya Ministry of Health (fever, *P. falciparum*–positive blood smear, and presentation with seizures or coma). The median age of patients with cerebral malaria was 3 years. Similar studies of clinical malaria are ongoing in several public and private hospitals in Homa Bay County.

## EFFECTIVENESS OF PRIMAQUINE ON *P. VIVAX* RECURRENCE IN ETHIOPIA

*Plasmodium vivax* blood-stage infection has the capacity to relapse weeks to years after mosquito transmission of sporozoites because of its dormant hypnozoite stage in the liver. *Plasmodium vivax* malaria relapses can be prevented by eliminating hypnozoites using primaquine, currently the only licensed drug for this purpose. In Ethiopia, *P. vivax* is co-endemic with *P. falciparum*, with the former accounting for approximately 35% of all cases of malaria.^54^ Recently, the Ethiopian National Malaria Strategic Plan added a 14-day course of low-dose primaquine to the chloroquine treatment regimen for radical cure of *P. vivax* malaria in low-transmission areas to facilitate elimination of transmission.^55^ Although randomized clinical trials in Ethiopia have documented the efficacy of primaquine in preventing *P. vivax* relapses under well-supervised conditions,^56^^,^^57^ the effectiveness of primaquine in real-world Ethiopia—where drug treatment adherence, stockouts, and parasite species misdiagnosis are common—is unknown. We conducted an observational study to investigate the effectiveness of 14 days of low-dose primaquine to prevent blood-stage *P. vivax* recurrence and reduce infectiousness to mosquitoes in the low-transmission Arjo study site. A cohort of patients was enrolled from multiple health facilities where stockouts of primaquine occurred frequently. Study participants attending health facilities with a reliable supply of primaquine typically received a standard treatment regimen of 25 mg/kg chloroquine for 3 days and a 14-day course of primaquine (0.25 mg/kg body weight daily) starting on day 2. Participants who attended health facilities with primaquine stockouts received only 3 days of chloroquine treatment. Preliminary analysis showed that adherence to a 14-day primaquine treatment regimen was difficult for many participants. A large proportion of participants who received the chloroquine–primaquine combination treatment showed recurrence of blood-stage *P. vivax* within the 6-month follow-up period.

## FUTURE PERSPECTIVES

Food insecurity and famine affect millions of residents of sub-Saharan Africa. Construction of dams and rural irrigation schemes has been widely recognized as key solutions to food security and economic development in drought-prone regions. Our field studies show that dams and irrigation increased the abundance of larval habitats, and enhanced habitat stability and productivity, subsequently leading to increased transmission and a greater malaria burden. Integration of hydrological models with remote sensing and malaria transmission models enhances the ability to predict how water-resource development projects affect vector larval habitats and malaria risks, thereby facilitating optimal water and environmental management practices and malaria control strategies.

Rapid spread of insecticide resistance, outdoor malaria transmission, emergence of invasive malaria vector species and new vector genotypes, and changes in vector ecology and malaria risk as a consequence of environmental modifications have limited significantly the effectiveness of current first-line interventions that target indoor biting and resting mosquitoes. New human settlement and migration associated with water-resource development also alter the landscape genetics of malaria parasites and vectors. Furthermore, the utility of intervention tools may vary because the effectiveness and cost of each tool differ among localities and over time. These complexities exemplify the need to identify interventions tailored to local conditions. To that end, our ICEMR research is currently conducting a cluster-randomized, sequential, multiple-assignment randomized trial to advance the development of optimal adaptive intervention strategies.^58^ Inter-sectoral collaboration among international academic institutions, development institutions, and government agencies in disease-endemic countries is crucial to the development and implementation of adaptive intervention strategies. We hope that our ICEMR research will catalyze such intersectoral collaborations to generate knowledge that informs policy and practices to improve malaria control and elimination efforts in the context of agricultural development projects in sub-Saharan Africa.
